# The short-term safety and effectiveness of a new distal perforating stent graft in Type B aortic dissection: a retrospective study

**DOI:** 10.1186/s12872-021-02270-5

**Published:** 2021-09-21

**Authors:** Xunqiang Liu, Wenkai Ji, Min Tian, Huanjun Chen, Cuihong Li, Liqiong Zhang, Ying Yang, Jifeng Wang, Min Ji, Chunxin Yang, Enshuai Zhu, Lei Cong, Xili Zhang, Xiaona Zhou, Hailong Liu, Jiaping Wang, Jing Tan, Jinhui Zhang

**Affiliations:** 1 Vascular Intervention Department, Yan’an Hospital Affiliated to Kunming Medical University, Kunming, China; 2 The First School of Clinical Medicine, Yunnan University of Chinese Medicine, Yunnan, China; 3 Pharmacy Department, Yan’an Hospital Affiliated to Kunming Medical University, Kunming, China; 4 Ultrasonic Department, Yan’an Hospital Affiliated to Kunming Medical University, Kunming, China; 5 Pain Treatment Department, First Affiliated Hospital of Kunming Medical University, Kunming, China; 6 Intervention Department, The Second Affiliated Hospital of Kunming Medical University, Kunming, China; 7 Office of Hospital Director, Yan’an Hospital Affiliated to Kunming Medical University, Kunming, China

**Keywords:** Thoracic endovascular aortic repair, Paraplegia, Type B aortic dissection

## Abstract

**Background:**

Spinal artery ischemia (SCI) events can result from over coverage of the descending thoracic aorta with a coated stent during Thoracic Endovascular Aortic Repair (TEVAR). The aim of this study was to determine whether a new distal perforating stent could reduce the incidence of spinal cord ischemia while remodeling the true lumen.

**Methods:**

TBAD patients treated with Talos stent in the vascular surgery Department of Yan 'an Hospital affiliated to Kunming Medical University between December 2017 and October 2019 were retrospectively analyzed to investigate the short-term safety and effectiveness of Talos stent.

**Results:**

A total of the 20 patients, including 14 males and 6 females, with an average age of 52.65 ± 8.98 years (range 37–68 years), were included in the analysis. Stent-grafts were successfully implanted in all patients under local anesthesia, with a technical success rate of 100%. The average operation time was 50.75 ± 13.01 min. A total of 2 cases (10%) presented chest pain associated with intercostal artery ischemia that was relieved on the 3rd and 5th postoperative day, respectively. Postoperative mean follow-up was 16.15 ± 3.99 months. No paraplegia or other complications occurred. And stenting did not induce new tears. No migration, deformation, or fracture of the stents occurred. There was a significant difference in the remolding of the true lumen preoperatively and at 12 months postoperatively (*P* < 0.05).

**Conclusions:**

Talos stent has achieved satisfactory clinical treatment results in short term.

## Background

Stanford B type aortic dissection (TBAD) is the type of dissection in which the first tear is located in the descending aortic. When the spinal cord, intercostal artery, gastrointestinal artery, renal artery, lower extremity artery, etc., are involved by the false lumen created by the dissection, the corresponding organs may be seriously affected due to loss of blood supply [1]. Currently, Thoracic Endovascular Aortic Repair (TEVAR) is considered the main therapy for the treatment of TBAD; however, the postoperative incidence of paraplegia is around 2–10% [2, 3]. Even in early clinical trials, some experts suggested that the stent's length should not exceed 205 mm to prevent postoperative spinal ischemic events. Although the risk of postoperative paraplegia could be reduced with the graft with limited length, continued false lumen flow due to the presence of distal reentry tear may result in true lumen collapse and may increase the likelihood of aortic adverse events.

**Fig. 1:**
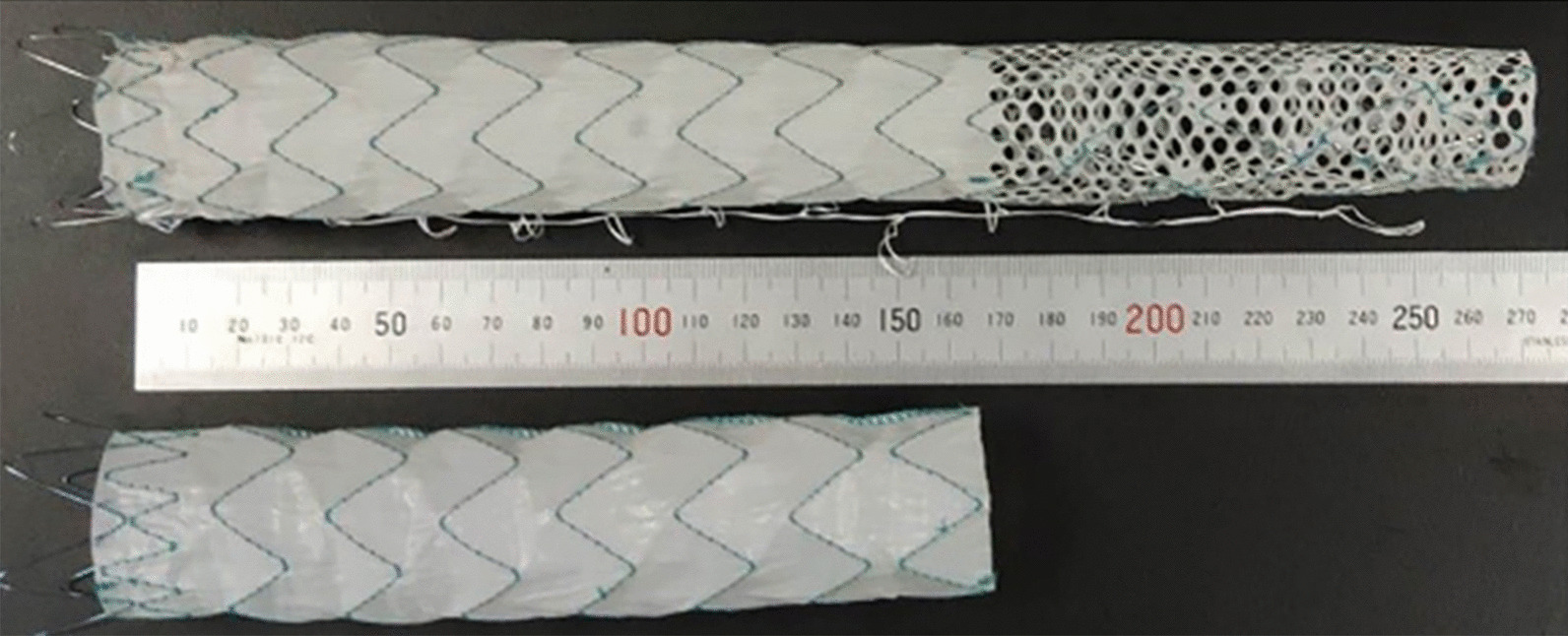
Comparison between Talos stent and other stents: Talos stent's unique distal perforating structure can enlarge the true lumen of the distal thoracic aorta and reduce the probability of spinal cord ischemia caused by covering the intercostal artery

Considering the above listed findings, a new stent graft (Talos stent, Shanghai Endovastec. Co., LTD., Fig. 1) was designed. The stent-graft with maximum 260 mm graft length can extend more distally so as to improve the remodeling of the distal true lumen. Also, the distal multi-perforated graft design ensures the blood supply to the intercostal arteries and spinal arteries, reducing postoperative paraplegia risk. The tapered size in diameter of the Talos stent conforms to the vessel's anatomy so as to reduce the stimulation to the blood vessel wall and avoid new tears caused by excessive expansion of the distal end of the stent.

In the present study, we retrospectively analyzed the clinical data of 20 TBAD patients treated with Talos stent in the Vascular Surgery Department of Yan 'an Hospital affiliated to Kunming Medical University between December 2017 and October 2019 to verify the short-term safety and efficacy of Talos stent.

## Methods

### Study design

The research procedures performed in this study were approved by Ethics Committee of Drug Clinical Trial in Yan'an Hospital, Kunming Medical University (IRB-PJ-2017-003). Informed consent for the use of a novel stent during treatment (Talos stent) and for the analysis of data published in the study was obtained from all the patients.

A total of 20 patients were retrospectively enrolled in the study. All the 20 patients of our center who had been previously treated for type B aortic dissection, are enrolled from the multicenter premarket clinical trial of Talos stent between 2017 and 2019. The inclusion criteria of the clinical trial was: (1) male or non-pregnant women aged between 18 and 85 years; (2) subjects diagnosed with TBAD; (3) the length of proximal landing zone ≥ 15 mm; (4) the subject has appropriate iliac and femoral artery approaches; (5) subjects judged to be suitable for endovascular or surgery treatment; (6) subjects who can understand the purpose of the study, voluntarily participate in and sign informed consent, and are willing to conduct follow-up. The exclusion criteria was: (1) subjects diagnosed with Stanford type A aortic dissection; (2) there was severe stenosis or calcification at proximal landing zone; (3) chimney tech was planned to be used to reconstruct branch artery; (4) subjects with Marfan's syndrome; (5) subjects judged to be unsuitable for endovascular treatment due to other reasons.

Patients were required to have had a baseline (first) postoperative computed tomography angioplasty (CTA) scan. The follow-up period was 6 months and 12 months after the operation. CTA examination was used to determine the surgical effect [4].

### Procedure

All the surgeons came from the same team and were experts with extensive experience in the field. After local anesthesia with 2% lidocaine was administered, a 6F arterial sheath (TERUMO RADIFOCUS introducer) was inserted through the left radial artery. Two vascular closure devices (Abbott Laboratories, Perclose ProGlide) were inserted through the guide wires, and the sutures were embedded, after which a 10F sheath (TERUMO RADIFOCUS introducer) was then placed. A 5F standard pigtail catheter (Cordis, Angiographic Catheter) was introduced and maintained in the ascending aorta through the left radial artery sheath. A 5F gold-labeled pigtail catheter (COOK, Centimeter Sizing Catheter) was advanced and kept in the ascending aorta through the femoral artery sheath.

Ascending aorta angiography was performed via an ordinary pigtail catheter to verify the anatomy of the aortic arch and its branches, as well as the location of the first tear. The proximal diameter of the aorta was measured by a gold-labeled pigtail catheter from the femoral artery. The gold-labeled catheter was exchanged by a super-stiff guidewire (COOK Lunderquist). Talos stent was advanced through the super stiff guidewire when the tip of the delivery system was in the aortic arch, and the outer sheath was removed. The proximal end of the stent-graft was positioned to the desired location, verified by DSA. The delivery system was held stationary, and the control panel was rotated clockwise to the limit, after which the yellow ring was quickly pulled to release the stent-graft. The control panel was rotated clockwise to the limit again, and the green ring was pulled to release the bare stent. Finally, the control panel was rotated clockwise to the limit once again, and the connector was pulled back at the end of the inner tube until the tip of the delivery system retreated back into the outer sheath.

Digital Subtraction Angiography (DSA) was performed to confirm the angiographic results. DSA was performed in the distal multi-perforated part of the stent. All patients were assessed for pain intensity by visual analogue scale (VAS) at 5 days postoperatively. VAS is widely used in clinical practice worldwide as well as in China to assess pain from subjective view [5]. We used a ruler with a length of about 10 cm, where one side was marked with 10 scales, and the two terminals were marked with "0" and "10" points, respectively; 0 points indicated no pain, and 10 points indicated the most intense pain that was unbearable. Patients would choose the points based on their subjective feeling to the pain caused by dissection after treatment.

### Study outcomes

The study's outcomes were: (1) successful treatment at 12 months: no technical failure, or no secondary intervention during 12 months of follow-up. Technical failures included the failure of implantation, persistent type I and III postoperative endoleak, death within 24 h postoperatively, or secondary intervention for the unexpected treatment of aortic dissection. (2) Thirty days MAEs [6, 7]: (Major Advance Events) severe adverse events that occurred within 30 days or during hospitalization.

Device-related adverse events included access arterial complication, persistent fever of unknown cause, rupture and retrograded aortic dissection; type I or III endoleak; stent migration (over 10 mm), infection, fracture and VAS point.

### Follow-up

All patients underwent CTA examination before the operation and at 6, 12 months postoperatively to evaluate the variation in the diameters of the true and false lumen, as well as the morphology of the stent and treatment results during the follow-up. For variation in the diameters, measurements were made at 3 panels, which are bifurcation of the trachea, lower margin of the left atrium, and the upper margin of the celiac trunk artery.

### Statistical analysis

Continuous variables are presented as mean ± standard deviation. Categorical variables are stated as absolute numbers and proportions. Paired sample T-test was used to compare the diameter of the true lumen, the false lumen, and the total arterial diameter at the same level in different periods. SPSS 22.0 (IBM Corporation, Arkmont, NY) software was used for statistical analysis. A *P* value of < 0.05 was considered statistically significant.

## Results

### Patients

**Fig. 2:**
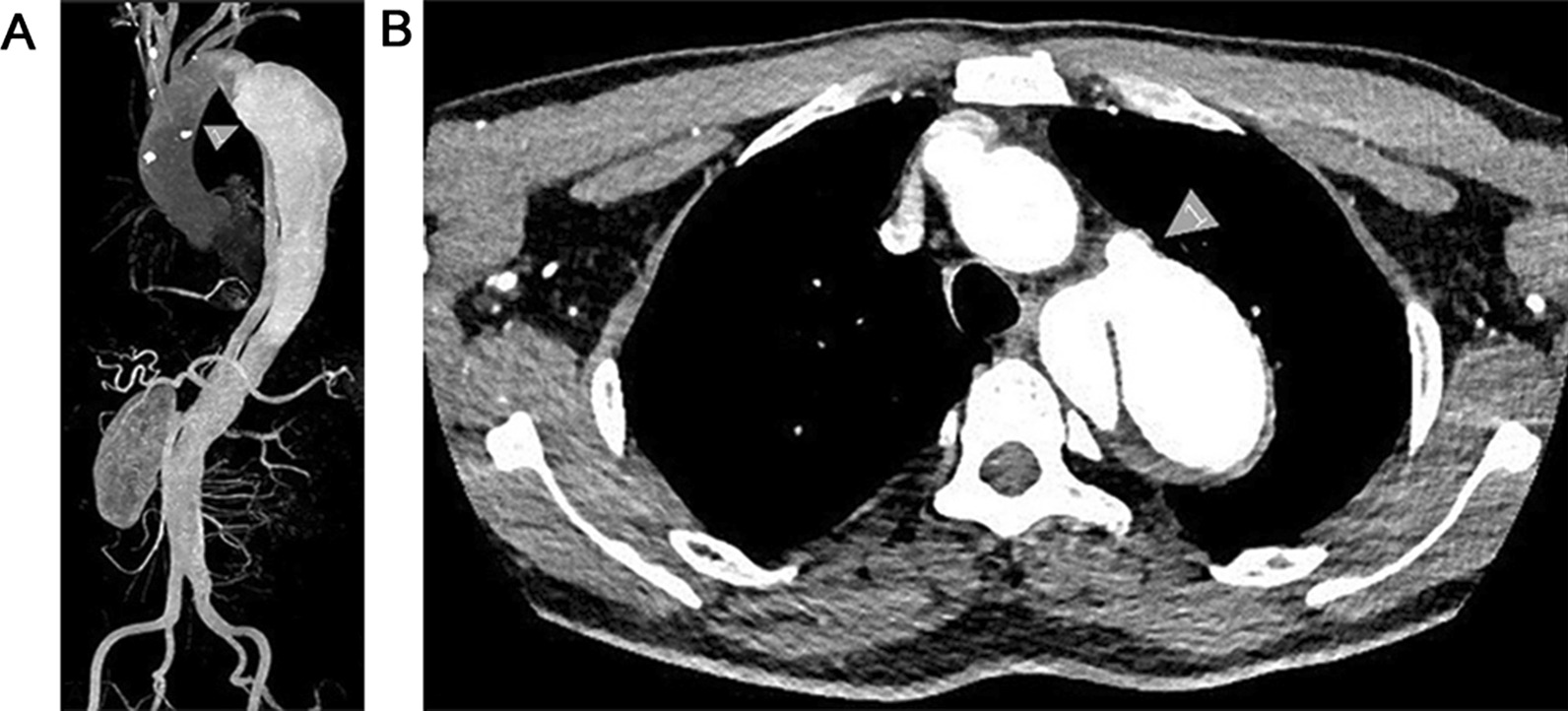
Preoperative CTA was used to determine the anatomical morphology of the patient's lesion. **a** It can be seen that the false lumen was huge and involved the whole descending thoracic aorta, and the true lumen was severely compressed. **b** Arrow 1 marked the false lumen

During the study period, 20 patients with the Talos stent, 14 males and 6 females with an average age of 52.65 years ± 8.98 years, were enrolled. The comorbidities are shown in Table 1. At the time of admission, all of them received antihypertensive treatment. Among 20 enrolled patients, 19 patients were diagnosed with acute TBAD with chest, back, waist, or abdomen pain for 1–6 days, and 1 patient was diagnosed with chronic TBAD with acute back pain 18 days ago but gradually subsided into dull pain. Of the 20 patients, 1 had a superior mesenteric artery dissection, 1 had a celiac artery dissection and splenic artery occlusion, 1 had a right iliac artery occlusion, and 1 had a left iliac artery occlusion. Aortic CTA (computed tomography angioplasty) was performed before the operation to identify the location, size, and extent of involvement of the aortic dissection (Fig. 2) and to determine the treatment plan.

**Table 1:** Demography data of patients

Indication	Result (n = 20)
Male	14 (70%)
Age (year)	52.65 ± 8.98 (37–68)
*Anamnesis*	
Hypertension	19 (95%)
Scoliosis	1 (5%)
Pleural effusion	6 (30%)
Pulmonary infections	17 (85%)
Hepatic and renal insufficiency	1 (5%)
Heart disease	1 (5%)
*Distal involvement*	
Thoracic aorta	2 (10%)
Abdominal aorta	12 (60%)
Below abdominal aorta	6 (30%)

### Overall outcomes

The Talos stent was successfully implanted in all the 20 patients, and the technical success rate was 100%. In 18 patients, the length of the proximal covered graft was 160 mm, the length of the distal multi-perforated graft was 60 mm in 14 patients and 100 mm in the other 4 patients. In 2 patients, the length of the proximal covered graft was 200 mm, the length of the distal multi-perforated graft was 60 mm. The stent specifications for all patients are shown in Table 3. The left subclavian artery was preserved in 8 patients and covered in 1 patient, who’s right vertebral artery was the dominant artery. The proportion of the distal multi-perforated graft in the total length of the stent ranged from 23.0 to 38.4%.

The mean operative time was 50.75 ± 13.01 min. VAS (visual analogue scale) score of intercostal artery-related chest pain was 1.50 ± 1.61 points on the 5th day postoperatively. Table 2 describes detailed information about the operation.

**Table 2:** Perioperative data of patients

Indication	Result
Operation time (min)	50.75 ± 13.01 (30–80)
Postoperative hospital stay (days)	12.4 ± 2.9 (8–19)
VAS score 5 days after surgery	1.50 ± 1.61 (10–5)
Postoperative intercostal artery (strip)	4.90 (1–10)

**Fig. 3:**
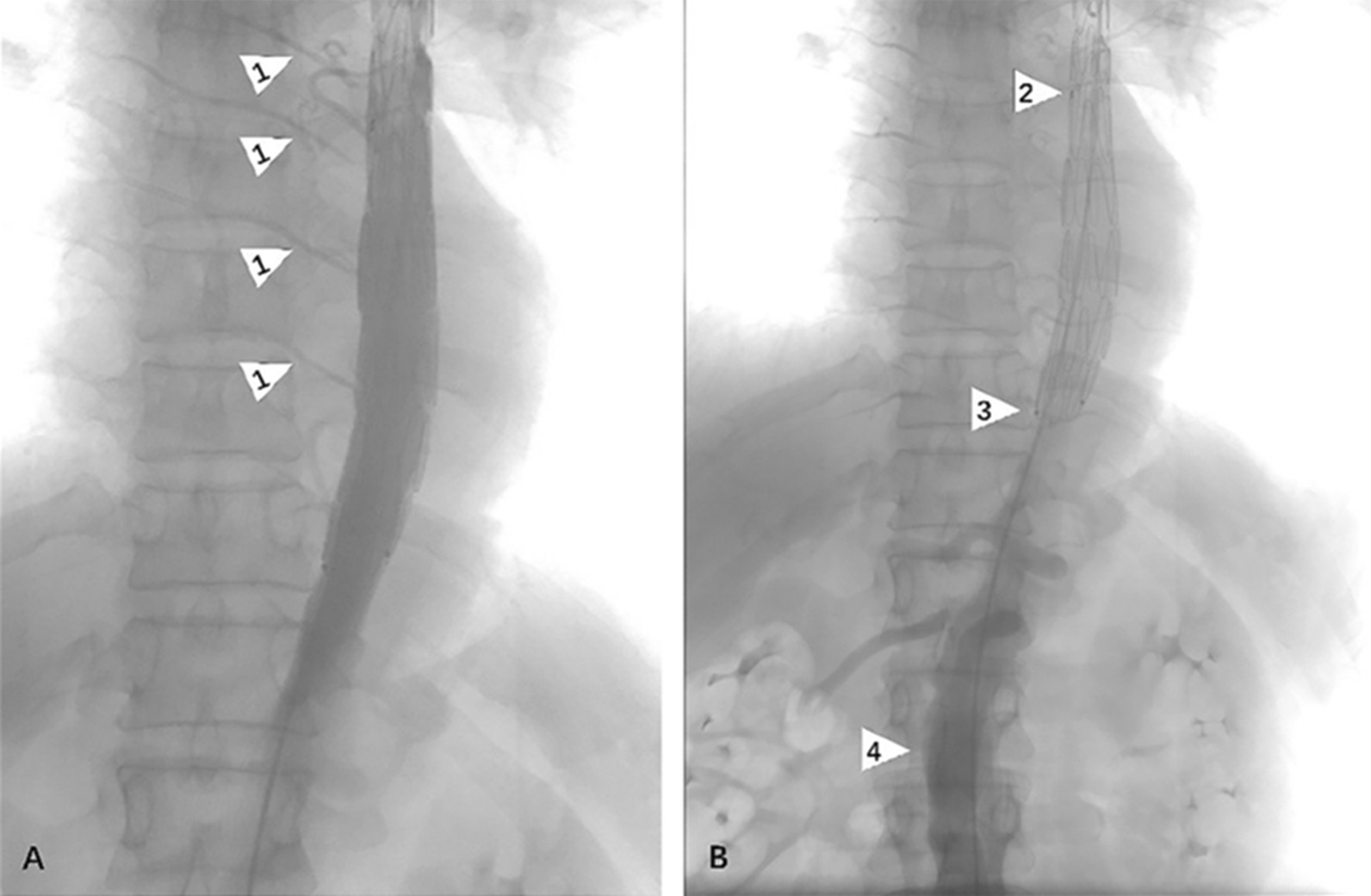
Angiography was performed immediately after stent implantation to confirm blood supply to the distal end of the stent. **a** Arrow 1 indicates the intercostal artery. **b** Arrow 2 indicates the junction of the coated section and the perforated section; Arrow 3 is the distal end of the entire stent and arrow 4 is the abdominal aorta

Postoperative DSA showed that Talos stent presented satisfactory conformity to the aortic wall without type I or III endoleak. In the distal multi-perforated graft part of the stent, intercostal arteries were well developed as well as the true distal lumen and branched arteries (Fig. 3). The number of intercostal arteries ranged from 1 to 10 with an average of 4.90.

Table 3 describes the specification of the stent. Postoperative mean follow-up was 16.15 ± 3.99 months. A total of 2 cases (10%) presented chest pain associated with intercostal artery ischemia, which was relieved on the 3^rd^ and 5^th^ postoperative days. During the follow-up, the incidence of paraplegia and endoleak rate were 0%. No migration, deformation, or fracture of the Talos stent occurred.

**Table 3:** Stent implantation specifications and follow-up time

Nos	Stent-graft		Maximal VAS Score	Postoperative intercostal arteries (pieces)	Follow-up (month)
	Cover length (mm)	Perforate length (mm)			
1	160	60	0	1	22
2	160	60	0	6	22
3	160	60	2	2	21
4	160	100	1	8	21
5	160	60	0	2	21
6	160	60	4	5	20
7	160	60	2	8	19
8	160	60	1	4	18
9	160	100	3	10	17
10	160	60	3	2	16
11	160	60	4	3	12
12	160	60	0	7	12
13	160	60	0	5	12
14	200	60	0	6	13
15	160	60	2	3	12
16	200	60	0	4	15
17	160	60	2	3	14
18	160	60	1	4	12
19	160	100	5	7	12
20	160	100	0	8	12
	164	68	1.51 ± 1.61	4.90 (1–10)	16.15 ± 3.99 (12–22)

VAS, Visual Analogue Scale

The 12-month treatment success rate was 100%. The incidence of MAE at 30 days was 0%. The 30-day mortality rate was 0%. The 12 months type I or type III endoleak rate was 0%, the incidence of paraplegia was 0%, and the frequency of device-related adverse events was 0%.

### Complete thrombosis

**Fig. 4:**
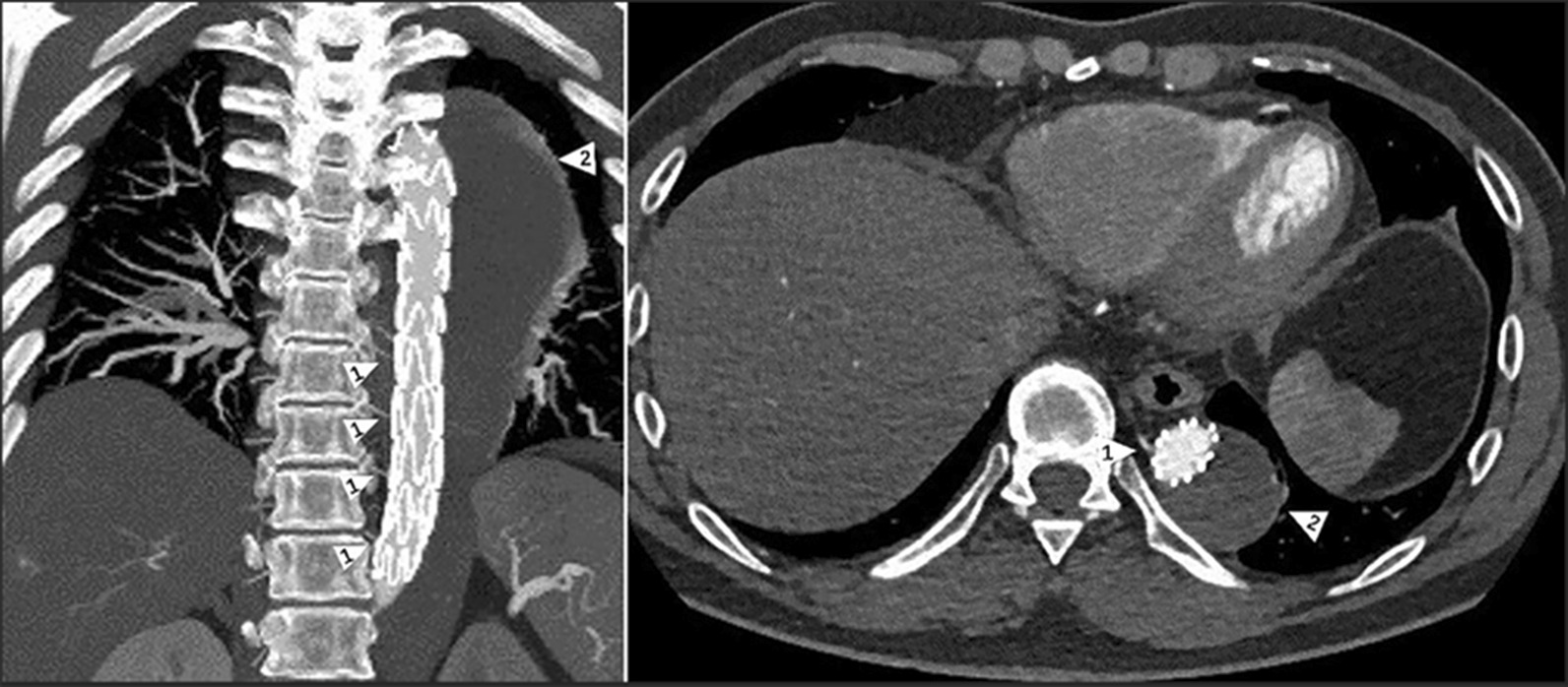
CTA review postoperative 1 week: Talos stent completely isolated the false lumen, and the blood supply of the intercostal artery was patched. Arrow 1 indicates the intercostal artery and arrow 2 indicates the false lumen

Among 20 patients, 6 patients had the disappearance or complete thrombosis of the false lumen, where 5 patients had no tear in the aorta distal to the stent graft and achieved entire repair of the dissection (Fig. 4). Thirteen patients had complete thrombosis of the false lumen in the descending aorta covered by the stent graft; 1 patient still had 2 tears in the aorta, among which 1 tear was at 102 mm above the celiac trunk artery, and the other was at the origin of the superior mesenteric artery.

**Fig. 5:**
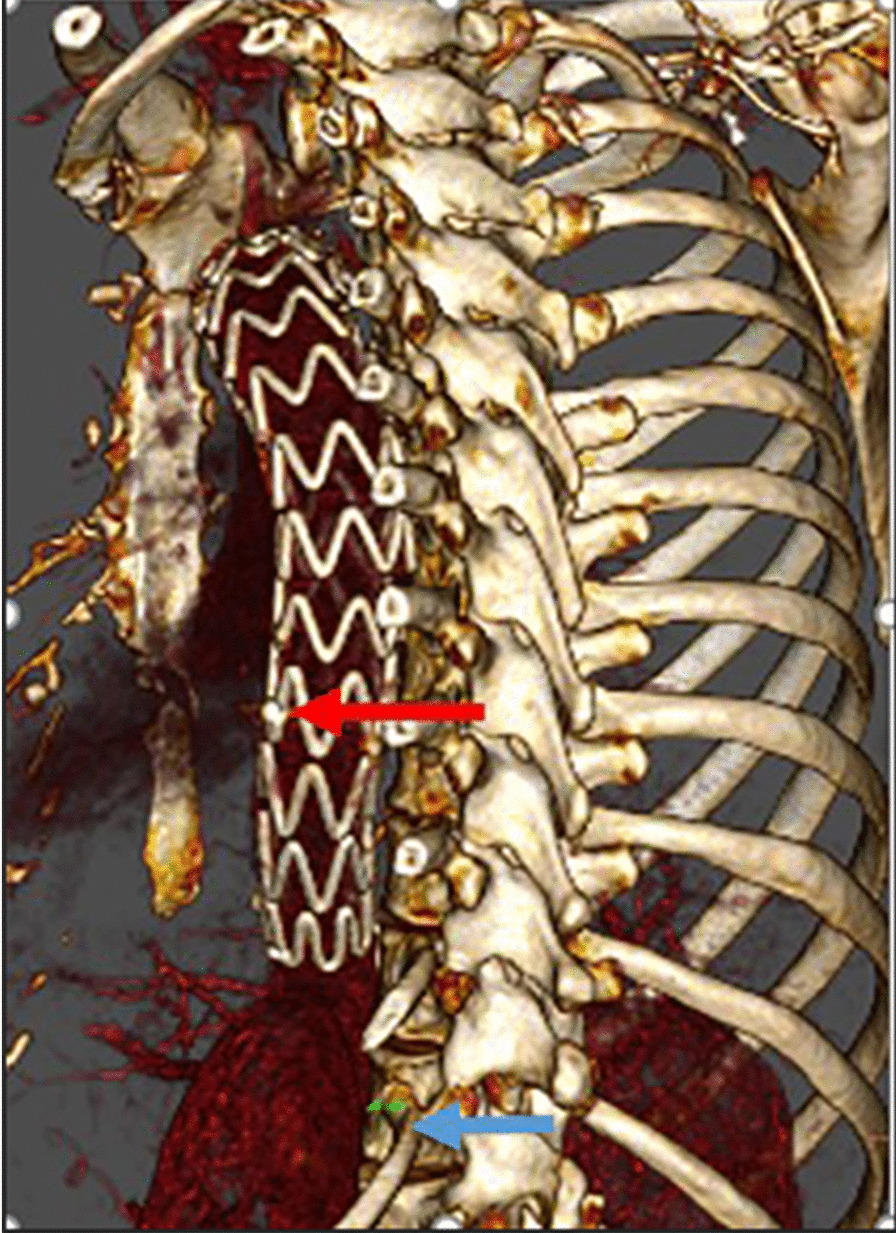
Evaluation of the marker indicating the starting position of the multi-perforated segment and thoracic vertebra. All patients were evaluated through the 3D reconstruction of CTA postoperative 12 months. The red arrow points to marker, and the blue arrow points to T12

According to observing the relative position between the marker and thoracic vertebrae, we found that 40% (8 cases) of marker was flushed with T9, and 55% (11 cases) of marker was flushed with T8. Only 5% (1 case) marker was level with T10, as shown in Fig. 5.

### Dissection involving the abdominal aorta

Among 20 patients, 18 of them had dissection involving abdominal aorta. The false lumen thrombosed rate in 6 and 12 months follow up is shown in Table 4. For the other 2 patients with the constrained false lumen at descending aorta, full thrombosis was found in 1 patient.

**Table 4:** The false lumen thrombosed rate of patients with dissection involving abdominal aorta

Thrombosed area	Postoperative 6 months	Postoperative 12 months
Above the trachea bifurcation	50.0% (9/18)	88.9% (16/18)
Above the lower margin of the left atrium	44.4% (8/18)	50.0% (9/18)
Above the upper margin of the celiac trunk artery	11.1% (2/18)	27.8% (5/18)

In the plane of trachea bifurcation, the true lumen remolding and false lumen shrinkage were statistically significant at different time periods. The whole aorta's significant shrinkage was only observed in comparing diameters from preoperative and postoperative 12 months.

At the lower margin of the left atrium, the diameter of the whole aorta was maintained stable throughout the follow-up period. There was significant improvement in true lumen remodeling and false lumens reduction between preoperative CTA and at 6 and 12 months follow-up. Comparing 6 month and 12-month CTA, only false lumen reduction was significant.

**Fig. 6:**
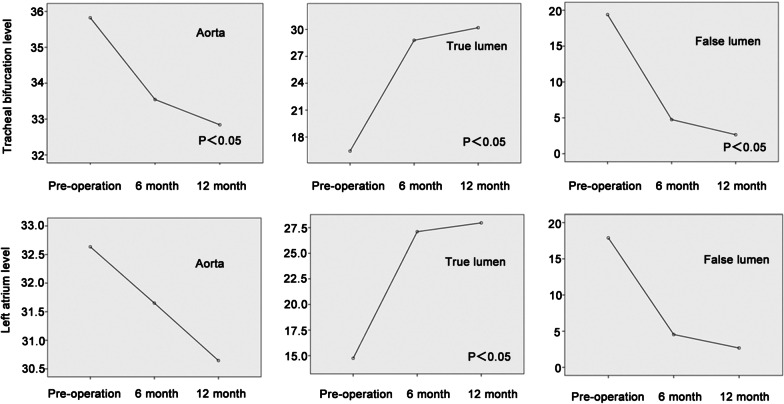
Univariate repeat ANOVA was used to demonstrate the active lumen diameter changes at the tracheal bifurcation level and the left atrium level. It can be seen that after stent implantation, the true lumen enlargement and false lumen contraction in different planes were statistically significant

At the level of the celiac trunk, the whole aorta significantly shrunk after postoperative 12 months. The trend of true lumen remodeling was significant between preoperative CTA and at 6, 12 months follow up. Comparing 6 month and 12 month CTA, only the true lumen remodeling showed significant improvement (Fig. 6).

### Tears in the abdominal aorta

Among the 14 patients with preoperative tears in the abdominal aorta, 2 patients had tears at 20–60 mm above the celiac trunk artery, 4 patients had tears at 5–20 mm above the celiac trunk artery; 5 patients had tears originated from the celiac trunk artery, and 1 patient had a tear in the abdominal aorta 20 mm below the renal artery. We calculated the changes of the true and false lumen and the diameter of the aorta in 14 patients preoperatively and 12 months postoperatively at the level of the celiac trunk, confirming that the dilation of the artery and true lumen were statistically significant, but the reduction of the false lumen was insignificant, as shown in Table 5 described.

**Table 5:** Diameter changes and statistical analysis of the celiac trunk level between preoperative and postoperative 12 months

Measurement level (mm)	Preoperative	Postoperative 12 months	*P* value
Aorta	28.75 ± 4.24	30.39 ± 3.60	0.030
True lumen	13.12 ± 4.08	16.75 ± 3.96	0.002
False lumen	15.63 ± 6.59	13.64 ± 5.60	0.161

## Discussion

Clinically, TBAD patients with a large false lumen and a compressed narrow true lumen are at high risk of dying due to rupture of the aorta or severe ischemia of the internal organs [1]. At present, TEVAR has become the accepted mainstream treatment for acute TBAD due to the advantages, such as minimal invasion, fast recovery, and fewer complications [2]. However, due to the wide extension of dissection and limitation related to stent length, patients with dissection involving the distal thoracic aorta often do not benefit from TEVAR. In their study, Kammam et al. [8] have confirmed that complete thrombosis of the false lumen is associated with improved 5-year survival rate postoperatively. Some experts [9, 10] pointed out that an incomplete seal of the distal tear and postoperative endoleak will lead to the incomplete remolding of the true lumen. With the increase of cases of TBAD treated by TEVAR, issues related to the incomplete thrombosis of the distal false lumen and persistent narrow in the true distal lumen are getting increasing clinical attention. He et al. [11] have shown that distal implantation of restricted bare stents can effectively expand the true lumen and promote the false lumen's thrombosis. Nevertheless, adverse events of bare stents also limit its further application. Melissano et al. [12, 13] reported a case of aneurysm rupture due to bare-metal stents. Hofferberth et al. [14] reported a case with bare-metal stent migration, which eventually resulted in re-intervention and death. Moreover, the bare stents currently available in the market have a relatively large diameter (36–46 cm), which may cause new distal aortic injuries [15].

Talos stent aims to improve the remodeling of the true distal lumen by extending the length of the stent-graft. Also, the distal part of the graft is multi-perforated in order to decrease the paraplegia rate, which the longer coving length may cause by graft. The change in diameter of the true and false lumen at six months and 12 months showed that sustained enlargement in the true lumen and the shrinkage in the false lumen were statistically significant after stent implantation, thus indicating satisfactory remodeling of the true lumen (Fig. 6). Six patients (30%) achieved the disappearance or complete thrombosis of the false lumen; 13 (65%) patients had complete thrombosis of the false lumen in the descending aorta covered by the stent-graft. Among the 20 patients, 14 patients still had tears in the abdominal aorta, which could not be covered by stent-graft and remain to be resolved. Nonetheless, the changes in the diameter of the whole aorta and true lumen in the celiac trunk of the 14 patients achieved statistical significance (Fig. 4), thus confirming the effectiveness of the Talos stent in aortic remodeling. The distal thoracic true lumen remodeling helps to provide more blood flow to the abdominal aorta and viscera, thus decreasing the relevant complications, including acute renal failure, intestinal ischemia, and similar. In our study, CTA review at postoperative 12 months showed no significant changes in abdominal lesions, while the incidence of complications caused by the remaining tears and dissection in the abdominal aorta was 0%, which requires close observation and follow-up.

It has been proved that the extended coverage of the descending aorta is one of the critical risk factors for spinal cord ischemia after TEVAR [16–18]. Feezor et al.[19] reported that the coverage length with spinal cord ischemia was 17.3 ± 21.8 cm (*P* = 0.0006). Amabile et al. [3] reported that 20.5 cm was the threshold of aortic covering length, which may lead to increased risk of spinal cord ischemia after TEVAR. The above results limit the application of longer stent-graft length in TEVAR. In order to solve the problem of too many spinal arteries being covered, Talos stents innovatively developed a stent structure with distal perforation, which ensured the blood supply from the distal thoracic aorta to the spinal artery with sufficient reopening of the true lumen.

The Adamkiewiecz artery is the most critical artery supplying the 4th spinal segment, 75% of which originates from T9 to T12 [20]. In our study, Talos stents selected for the 20 patients were 232 mm in the whole length with an average 164 mm in graft length and an average 68 mm in distal multi-perforated graft length. Based on the observation of the relative positions of the markers, which identified the starting position of the multi-perforated segment and thoracic vertebrae in 20 cases, 40% of the markers were in line with T9, 55% of the markers were in line with T8, and 5% (1 case) were at the level of T10. The position of the multi-perforated segment was between T8 and T10, which can help reduce the incidence of postoperative paraplegia by avoiding covering the Adamkiewiecz artery. Postoperative angiography showed that average of 4.9 intercostal arteries was preserved in 20 patients, and 2 patients experienced chest pain, which was relieved before discharge. The incidence of paraplegia was 0% at 12 months follow-up. We hope that future studies will recruit more patients to further verify the clinical efficacy of Talos stent in reconstructing intercostal arteries and preventing spinal cord ischemia events.

All the selected stents are tapered in diameter, whose distal diameter is 6 mm smaller than the proximal one. For acute TBAD, the proximal diameter of the stent is usually 5–10% larger than the proximal diameter of the aorta [21], while for chronic TBAD, the increase rate in diameter should be 20% [22], which makes the distal diameter of the stent-graft much larger than the true distal lumen, and the ratio is often higher than 120% [23], resulting in unreasonably higher radial force to the fragile aortic wall of dissection and causing a new tear or pseudoaneurysm at the distal thoracic aorta [23]. The tapered size in diameter (Fig. 1) may improve the conformability to the anatomy and reduce relative complications.

### Limitation

This study was a single-center retrospective study, and only 20 patients were followed up for one year after surgery. In terms of data analysis, the sample size may be too small, which may lead to deviations in conclusion. In our future studies, we will further expand the number of cases and follow-up time and actively carry out prospective studies to further verify the reported findings.

## Conclusion

Talos stent-graft with distal multi-perforated graft in design has achieved satisfactory clinical treatment results for TBAD within 12 months, especially in promoting the remolding of the true distal lumen and preserving the distal intercostal arteries and spinal arteries, which can help provide sufficient blood flow to the abdominal aorta, visceral organ, and lower extremity and decrease the incidence of paraplegia. Nonetheless, the safety and effectiveness in the medium and long term need further follow-up, and more cases should be enrolled.

## Data Availability

The datasets used and/or analysed during the current study are available from the corresponding author on reasonable request.

## Abbreviations

SCI: Spinal artery ischemia
TEVAR: Thoracic endovascular aortic repair
TBAD: Type aortic dissection
CTA: Computed tomography angioplasty
DSA: Digital subtraction angiography
VAS: Visual Analogue Scale
